# Evaluation of MicroScan Bacterial Identification Panels for Low-Resource Settings

**DOI:** 10.3390/diagnostics11020349

**Published:** 2021-02-19

**Authors:** Sien Ombelet, Alessandra Natale, Jean-Baptiste Ronat, Olivier Vandenberg, Liselotte Hardy, Jan Jacobs

**Affiliations:** 1 Department of Clinical Sciences, Institute of Tropical Medicine, 2000 Antwerp, Belgium; lhardy@itg.be (L.H.); jjacobs@itg.be (J.J.); 2 Immunology & Microbiology Department, KU Leuven, 3000 Leuven, Belgium; 3 Médecins Sans Frontières, Operational Center Paris, 75019 Paris, France; alessandra.natale@paris.msf.org (A.N.); jean-baptiste.ronat@paris.msf.org (J.-B.R.); 4 Team ReSIST, INSERM U1184, School of Medicine, University Paris-Saclay, 94807 Villejuif, France; 5 Bacteriology-Hygiene Unit, Assistance Publique—Hôpitaux de Paris, Bicêtre Hospital, 94270 Le Kremlin-Bicêtre, France; 6 Center for Environmental Health and Occupational Health, School of Public Health, Université Libre de Bruxelles (ULB), 1050 Brussels, Belgium; olivier.vandenberg@ulb.ac.be; 7 Innovation and Business Development Unit, Laboratoire Hospitalier Universitaire de Bruxelles—Universitair Laboratorium Brussel (LHUB-ULB), Université Libre de Bruxelles (ULB), 1050 Brussels, Belgium; 8 Division of Infection and Immunity, Faculty of Medical Sciences, University College London, London WC1E 6BT, UK

**Keywords:** clinical bacteriology, low-resource settings, bacterial identification, blood cultures, MicroScan identification system

## Abstract

Bacterial identification is challenging in low-resource settings (LRS). We evaluated the MicroScan identification panels (Beckman Coulter, Brea, CA, USA) as part of Médecins Sans Frontières’ Mini-lab Project. The MicroScan Dried Overnight Positive ID Type 3 (PID3) panels for Gram-positive organisms and Dried Overnight Negative ID Type 2 (NID2) panels for Gram-negative organisms were assessed with 367 clinical isolates from LRS. Robustness was studied by inoculating Gram-negative species on the Gram-positive panel and vice versa. The ease of use of the panels and readability of the instructions for use (IFU) were evaluated. Of species represented in the MicroScan database, 94.6% (185/195) of Gram-negative and 85.9% (110/128) of Gram-positive isolates were correctly identified up to species level. Of species not represented in the database (e.g., *Streptococcus suis* and *Bacillus* spp.), 53.1% out of 49 isolates were incorrectly identified as non-related bacterial species. Testing of Gram-positive isolates on Gram-negative panels and vice versa (*n* = 144) resulted in incorrect identifications for 38.2% of tested isolates. The readability level of the IFU was considered too high for LRS. Inoculation of the panels was favorably evaluated, whereas the visual reading of the panels was considered error-prone. In conclusion, the accuracy of the MicroScan identification panels was excellent for Gram-negative species and good for Gram-positive species. Improvements in stability, robustness, and ease of use have been identified to assure adaptation to LRS constraints.

## 1. Introduction

### Bacterial Identification in Low-Resource Settings

Clinical bacteriology laboratories equipped to diagnose bacterial infections and perform antibiotic susceptibility testing (AST) are scarce in low-resource settings (LRS) [1,2]. Correct identification of bacteria in clinical samples is a cornerstone for proper management of bacterial infections because both empirical and directed antibiotic treatments depend on the identified organism [3,4].

Current “gold standard” identification methods in high-resource settings, such as matrix-assisted laser desorption/ionization time-of-flight mass spectrometry (MALDI-TOF MS) and molecular techniques are not adapted to diagnostic laboratories in LRS [1,5]. Most laboratories in LRS still rely on “conventional” phenotypic identification techniques, in which isolates are inoculated on different culture media containing different carbohydrates and enzyme substrates, and interpretation of test results is carried out using dichotomous decision trees [6,7,8]. Commercialized panels consisting of phenotypic tests, such as the Analytical Profile Index (API^®^) panels (bioMérieux, Marcy l’Etoile, France) offer probability-based identification; combined test results are compared to databases containing the profiles of thousands of bacterial isolates [9].

In response to the global rise in antimicrobial resistance and the need to implement context-tailored antibiotic stewardship programs, the non-governmental humanitarian organization Médecins Sans Frontières (MSF) committed to developing an all-in-one, affordable, and transportable clinical bacteriology laboratory, the Mini-lab [10]. Because the Mini-lab is intended for use in LRS by non-expert laboratory technicians, a system for bacterial identification is required that is easy to inoculate, easy to read, inexpensive, and with long shelf life. After defining technical specifications (target product profile) and performing a market analysis, the Dried Overnight MicroScan ID panels by Beckman Coulter (Brea, CA, USA) were chosen because they have a long shelf life and can be read without the use of automated instruments.

**Figure 1:**
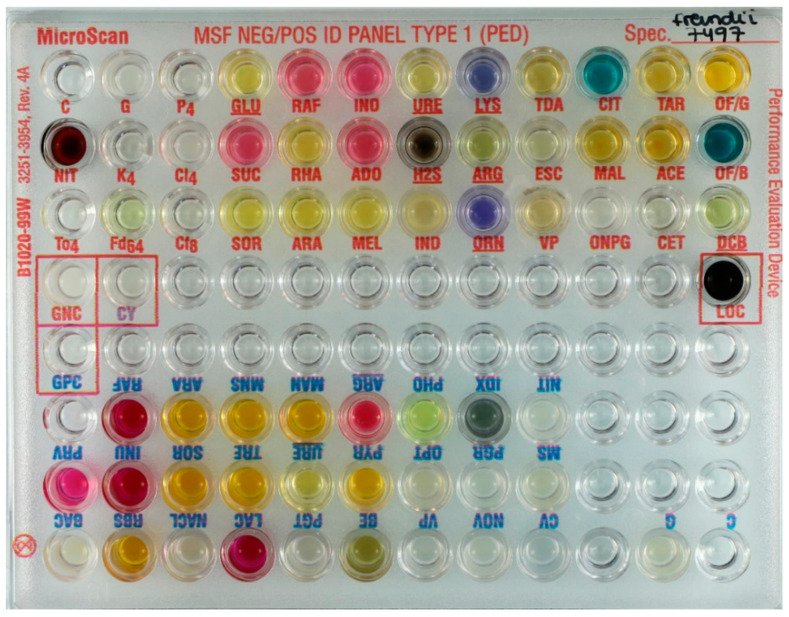
Example of an inoculated customized Médecins Sans Frontières (MSF) ID panel (MSFNPID1): Gram-negative test wells on the top side and Gram-positive test wells on the opposite side. Gram-positive and Gram-negative test panels can thus be inoculated simultaneously; for reading, one of the two sides must be picked.

To meet Mini-lab specifications, Beckman Coulter developed a customized “Research Use Only” ID panel containing all test wells for the identification of both Gram-negative and Gram-positive organisms on one single panel, coded MSFNPID1. The Gram-negative and Gram-positive test wells were assembled in separate groups on the panel, and therefore, knowledge of the Gram stain result is still essential to read the panel correctly (Figure 1). The primary objective of this study was to assess the diagnostic accuracy of these identification panels with clinical isolates originating from LRS. A secondary objective was to assess the ease of use of the system.

## 2. Materials and Methods

### 2.1. Identification Panels

We evaluated the Dried Overnight Positive ID Type 3 (PID3) and Dried Overnight Negative ID Type 2 (NID2) panels and the customized version of the MSF Negative Positive ID Type 1 (MSFNPID1) panel. We will further use the terms “Gram-positive panels” and “Gram-negative panels” when referring to the composition of test wells. When dealing with the specific panels, we will use the terms PID3, NID2, and MSF ID panels, respectively. Table 1 demonstrates the different tests performed on each panel and reagents to be added. The Beckman Coulter LabPro software version 4.43 and the MicroScan autoSCAN-4 automated reader were used for this evaluation. Lot numbers of panels are listed in Supplementary Table S1.

### 2.2. Clinical Isolates and Reference Strains

A total of 367 isolates were tested (Table 2 and Table 3), including 332 anonymized clinical isolates and 35 reference strains corresponding to the most common bloodstream pathogens or contaminants in LRS. Of the clinical isolates, 66.9% originated from sub-Saharan Africa, 26.8% from Asia, 4.2% from South America, and 2.1% from Europe (See Supplementary Table S2 for details per species). Reference strains were obtained from the Belgian Sciensano (Scientific Institute of Public Health) (https://www.wiv-isp.be/QML/activities/external_quality/rapports/_nl/rapports_annee.htm; accessed 18 February 2021) as part of external quality assessment programs. The collection included 323 isolates (195 Gram-negative and 128 Gram-positive) of species represented in the MicroScan database. The remaining 44 isolates, belonging to species that were not present in the MicroScan database, were used to look for anticipated incorrect identifications.

### 2.3. Reference Identification of Clinical Isolates

Identification of clinical isolates was confirmed by MALDI-TOF MS, using Microflex™ device (Bruker Daltonics, Billerica, MA, USA) with MALDI Biotyper^®^ (MBT) 7854 MSP Library. Serotyping of *Salmonella* was performed with the *Salmonella* antisera of Pro-lab diagnostics (Richmond Hill, ON, Canada). Optochin disks (Rosco Diagnostica, Taastrup, Denmark) were used to distinguish between *Streptococcus pneumoniae* and other viridans *Streptococcus* species, after identification by MALDI-TOF as *Streptococcus pneumoniae/mitis/oralis/parapneumoniae*.

### 2.4. Preparation of Suspension

Frozen isolates (stored at −80 °C) underwent two passages on sheep blood agar. Inoculation from single colonies was conducted using the Prompt™ Inoculation System (Beckman Coulter, Brea, CA, USA), consisting of a wand designed to hold a specific quantity of bacterial material and a 30 mL Prompt™ Inoculation Water bottle (Beckman Coulter, Brea, CA, USA). As per the manufacturer’s instructions, some slow-growing *Streptococcus* species were inoculated using the turbidity standard method, in which a 0.5 McFarland solution was prepared in 3 mL of Inoculum Water, of which 100 µL was pipetted into 25 mL of Inoculum Water.

### 2.5. Inoculation of Panels

The bacterial suspension was transferred to the panels using MicroScan Renok (inoculating device by Beckman Coulter, Brea, CA, USA), which delivers 115 µL of broth suspension to each well. Mineral oil was added to the indicated wells (Table 1). Panels were incubated in an atmospheric incubator for 16–18 h at 35 °C.

### 2.6. Reading of the Panels

**Figure 2:**
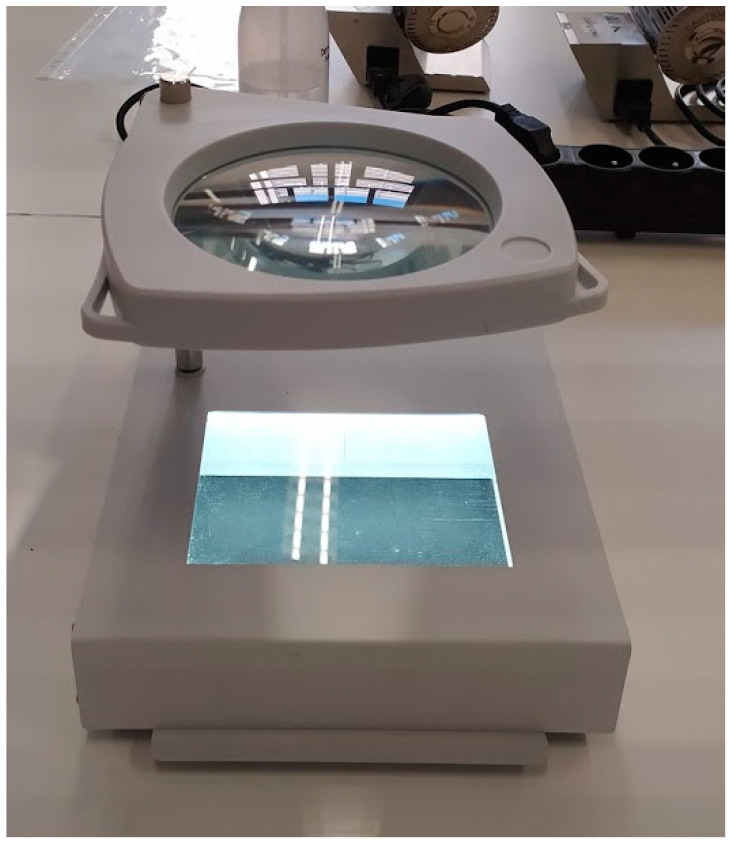
Prototype of the microplate viewer by JP Selecta, used for the visual reading. The background at the bottom can be changed to white.

Reagents were added and panels were read visually using transmitted light and magnifier with interchangeable white or black backgrounds according to the manufacturer’s instructions for use (IFU). We used a microplate viewer prototype adapted for this purpose by JP Selecta (Barcelona, Spain) (Figure 2). The results were recorded on PID3 and NID2 worksheets (supplied by the manufacturer) and transferred to the LabPro software (version 4.43). The LabPro software calculated the “biotype”, a code containing the biochemical profile, and generated the corresponding identification for each biotype with probability scores. Results with high probability scores (≥85%) were considered reliable, while results with low probability scores (<85%) were considered “unconfirmed”, as defined by the IFU. No additional tests for confirmation of low probability identifications were performed in our study. If the biochemical profile did not match any identification in LabPro’s database, the result generated was “very rare biotype”.

### 2.7. Robustness Testing

Incorrect panel: To anticipate possible errors in Gram stain, we inoculated 64 Gram-negative isolates on Gram-positive panels and vice versa (80 isolates).

Incorrect algorithm: For Gram-positive organisms, the LabPro software asked to choose between the algorithms for Streptococcaceae (*Streptococcus* species and *Enterococcus* species (catalase negative) versus Micrococcaceae (*Micrococcus* species, *Staphylococcus* species, and *Listeria monocytogenes* (catalase positive). To assess the impact of errors in microscopy or catalase results, we compared the results of Gram-positive bacteria with both the Streptococcaceae and Micrococcaceae algorithm.

Hemolysis: For the Streptococcaceae, visual assessment of beta-hemolysis (complete hemolysis) on blood agar was part of the biotype. In many LRS, sheep blood agar is hard to come by, hence hemolysis patterns may be less reliable in these settings [11,12]. To assess the impact of hemolysis reading, we compared results for beta-hemolytic streptococci with hemolysis read as positive versus negative.

Oxidase: For Gram-negative organisms, the software required the result for oxidase testing. We used OxiSticks™ Oxidase Swabs (Hardy Diagnostics, Santa Maria, CA, USA) for this purpose. For a subset of *Stenotrophomonas maltophilia* isolates, oxidase testing was repeated with Bactident^®^ oxidase (Merck Millipore, Burlington, MA, USA).

### 2.8. Comparison between Automated and Visual Reading, Repeatability and Inter-Observer Agreement

The panels of 190 isolates were read both by visual and automated reading. To assess the “repeatability” of the panels, 31 isolates were tested in triplicate. Panels were inoculated from a single blood agar plate and processed and read simultaneously and by the same operator. “Inter-observer agreement” of visual reading was assessed for 50 isolates, subsequently read by two operators blinded to each other’s results. For the above comparisons, species agreement was defined as an identical species identification; biotype agreement was defined as an identical biotype, implying identical results for each separate test well.

### 2.9. Definitions of Correct and Incorrect Identifications

Correct identification was defined as concordance between the reference identification method and the identification given by MicroScan software up to the level provided by the MicroScan system (species level for most organisms, serotype level for certain *Salmonella* serotypes and genus level for *Micrococcus* species, for instance). We assessed the potential impact of incorrect identifications in the light of diagnostic and therapeutic consequences, as well as epidemiological significance.

For species not represented in the database or when panels were incorrectly used (e.g., Gram-positive isolate on Gram-negative panel), the expected “correct” identification by the MicroScan system was “very rare biotype”, indicating the system did not find sufficient agreement with expected biotypes of species available in the database. We will further refer to this result as “no identification possible”.

### 2.10. Management of Incorrect Identifications

For incorrect identifications with high probability scores, MALDI-TOF was repeated. If initial reference identification was confirmed, the MicroScan panel was repeated or included in the repeatability testing panel: if the isolate was correctly identified upon repeat MicroScan testing, we assumed technical error on first testing and accepted the second result. For repeatability testing, if at least two of three repetitions yielded again an incorrect result, the isolate was considered incorrectly identified. For *Streptococcus* species, all incorrect identifications were repeated using the turbidity standard method instead of the Prompt™ method according to the MicroScan panel IFU.

### 2.11. Ease of Use

Ease of use was assessed by surveying the operators for feedback on each of the components of the system. The readability level of the IFU was assessed using Flesch–Kincaid Grade Levels (https://www.online-utility.org/english/readability_test_and_improve.jsp; accessed 18 February 2021) [13].

## 3. Results

### 3.1. Results for Species Included in the MicroScan Database 

Gram-negative isolates: Gram-negative isolates represented in the MicroScan database were correctly identified with a high probability score in 92.3% of 195 isolates (Table 2). An additional 2.6% of isolates were correctly identified but with a low probability score. Enterobacterales were more accurately identified than non-fermenters, with correct identifications in 96.1% and 90.0%, respectively. Table 4 shows an overview of the incorrectly identified isolates. Most errors for Enterobacterales (6/9) were at the species level and had limited clinical relevance. Six out of 15 *Stenotrophomonas maltophilia* isolates tested oxidase positive with the OxiSticks™; this led to incorrect identifications as other non-fermenters in four isolates. Repeat oxidase testing with Bactident^®^ oxidase tests gave negative results for all isolates; the corrected biotypes all produced correct identifications with high probability scores.

**Figure 3:**
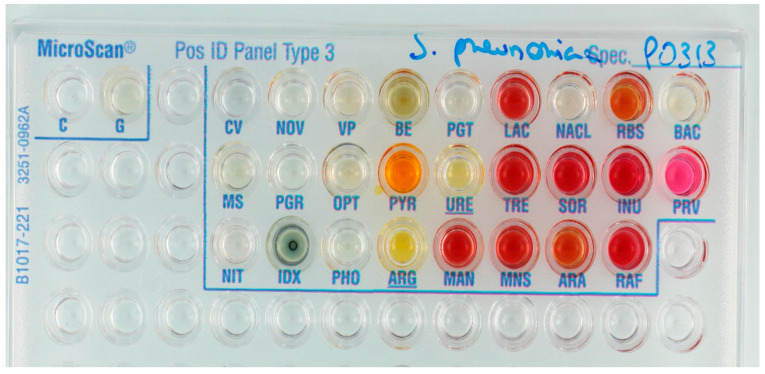
*Streptococcus pneumoniae* clinical isolate incorrectly identified as Gemella species; PNP-β-d-Glucuronide test (PGT) was considered negative (any shade of yellow = positive). A positive result for PGT would have led to correct identification of *S. pneumoniae* with a probability score of 94.79%. Only indoxyl phosphatase (IDX) test was positive in this panel.

**Figure 4:**
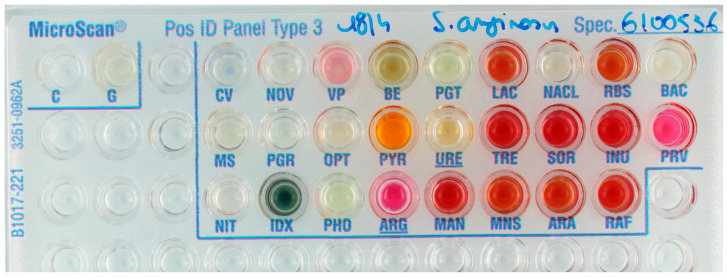
Streptococcus anginosus reference isolate incorrectly identified as S. mitis/oralis. Note that both PHO and PGT wells were considered positive when this panel was read; “any shade of yellow” leads to a positive result according to the instructions for use (IFU). A negative reading would have resulted in the correct identification of *S. anginosus*. Note also that Voges–Proskauer (VP) test was judged negative because “pale pink” is to be considered negative according to the IFU. A positive result of VP would have led to an identification of *S. parasanguinis*.

Gram-positive isolates: Of 128 Gram-positive isolates, 79.7% and 6.3% were correctly identified with high and low probability scores, respectively (Table 2). Low probability identifications were more frequent for Streptococcaceae than for Micrococcaceae (*Staphylococcus*, *Micrococcus*, and *Listeria* species), i.e., 8.3% of isolates versus 2.6%, respectively. Incorrect identifications were especially common—and considered as highly clinically relevant—for *Streptococcus pneumoniae* (3/12) and *Streptococcus anginosus* (4/8) (Figure 3 and Figure 4). All *S. aureus* isolates (*n* = 14) were identified correctly.

### 3.2. Isolates Not Represented in the Database

Testing of bacteria not represented in the MicroScan database led to incorrect high probability identification in 53.1% of 49 isolates tested, and only for 6.1% of isolates, the expected result “identification not possible” was generated (Table 4). The yeast species were consistently misidentified by the MicroScan Gram-positive panel as either *Staphylococcus cohnii* (*Micrococcus/Staphylococcus* algorithm) or *Rhodococcus equi* (*Streptococcus* algorithm) (Table 5).

### 3.3. Robustness Testing

Incorrect panel: Inoculating a Gram-positive isolate on the Gram-negative panel or vice versa led to an incorrect identification with a high probability score in 32.6% of 144 isolates tested (Table 6). This risk was higher for isolates wrongly inoculated on the Gram-negative panel (43.8%) compared to the Gram-positive panel (18.8%). A total of 38.2% isolates inoculated on the wrong panel generated the expected result “no identification possible”. *Rhodococcus equi* was a common incorrect identification with the Gram-positive panel (*n* = 27); when the Gram-negative panel was used for Gram-positive isolates, identification of *Providencia rustigiannii* recurred (*n* = 13) (Table 5).

Incorrect algorithm: When *Staphylococcus* species were interpreted erroneously using the *Streptococcus* algorithm, this led to incorrect high probability identification for 23.3% of isolates tested (Table 6). Vice versa, interpreting the results of *Streptococcus* species with the *Micrococcus* algorithm led to incorrect high probability identifications in 46.3% of isolates tested.

Hemolysis: Impact of hemolysis on identification of beta-hemolytic *Streptococcus* species (*S. agalactiae, S. pyogenes, S. dysgalactiae*) was limited; beta-hemolysis results led to different identifications in only 2 of 32 isolates (one *S. agalactiae* isolate and one *S. pyogenes* isolate).

### 3.4. Comparison between Automated and Visual Reading, Repeatability and Inter-Observer Agreement

Automated versus visual reading: Visual reading was slightly more accurate than automated reading (90.6% correct versus 87.5% correct), except for *Streptococcus* species. For species included in the MicroScan database, species agreement between visual and automated reading was 91.9%, and biotype agreement 58.1% (Supplementary Table S4).

Repeatability was better for automated reading than visual reading (89.7% versus 88.7% for species agreement; 87.5% versus 79.0% for biotype agreement). Especially for *Staphylococcus* species and Enterobacterales, repeatability was excellent with 100% biotype agreement for the automated reading. The lowest repeatability was seen for *Streptococcus* species (70.8% biotype agreement for automated reading versus 73.1% for visual reading).

Inter-observer agreement: On 50 isolates read by two operators, species and biotype agreements were 98.0% and 66.0%, respectively. VP (Voges-Proskauer test) was the test most often read differently by different observers (inter-observer and visual-automated) (Supplementary Table S5).

### 3.5. Ease of Use 

Instructions for use: The instructions for interpretation (e.g., color) of positive or negative results were often equivocal (Table 7). Flesch–Kincaid Grade Levels of the Gram-negative, Gram-positive, and MSF panel IFU were 9, 11, and 10, respectively. Flesch–Kincaid Grade Levels refer to US grade levels (i.e., years of schooling) necessary to understand the text.

Inoculation and reading: Both the Prompt™ and the RENOK system (Beckman Coulter, Brea, CA, USA) were considered user-friendly and time-efficient, especially compared to other inoculation methods using 0.5 McFarland standards and pipettes. Reading of the panels was considered rather difficult. Visual assessment of positivity of wells was frequently doubtful. A harmonized workflow for reading multiple panels was hindered by the re-incubation requirements; reagents could only be added to the panels when certain combinations of tests were positive—if this was not the case, re-incubation for another 24 h was needed. Furthermore, waiting times between adding reagents and visual reading were variable, ranging from immediate reaction to 20 min. Automated reading was more user-friendly but a visual check of the required positive wells was still necessary. Re-incubation of panels was needed for 15.3% of panels.

**Figure 5:**
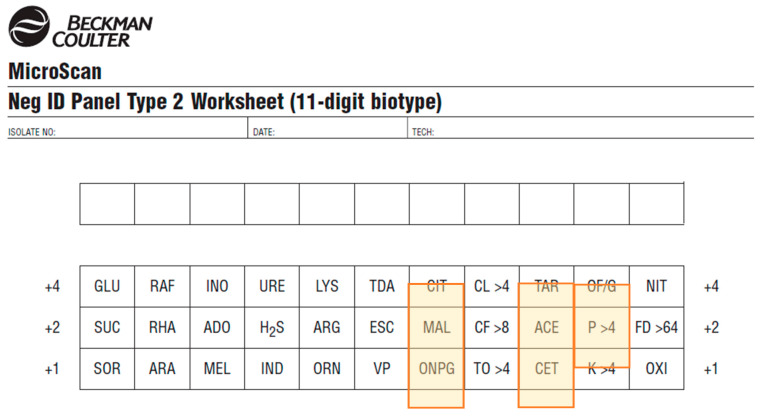
Worksheet for the NID2 panel. Tests marked in orange are in a different order on the panel than they are on the worksheet (see Figure 1).

Obtaining identifications: The worksheets provided with the PID3 and NID2 panels had some tests in different positions compared to the real-life panel or the software picture (Figure 5). To obtain identification results from the biotype, the LabPro software and a freely available online Biotype Lookup Tool could be used. 

Customized panels: Because Gram stain results were still necessary to interpret the results of the inoculated panels, advantages in the usability of the customized MSF ID panels were mainly logistic (simplicity, stock management). On the other hand, care should be taken when reading the panels to avoid reading the wrong side.

### 3.6. Stability

The shelf life of the panels was within the limits predefined by the Mini-lab target product profile (minimally 12 months); storage conditions for the panels (2–25 °C) were not entirely within the Mini-Lab specifications (2–40 °C). Packaging of individual panels was of good quality, providing air-sealed individual aluminum-plastic pouches with a humidity indicator. Other components were provided within sturdy cardboard boxes. The shelf life of the VP2 reagent (Voges Proskauer test) was too short; it could be used for just two weeks after reconstitution of the reagent.

## 4. Discussion

### 4.1. Performance of the MicroScan Panels

Our results showed excellent performance of the Gram-negative panel, with 94.9% of isolates correctly identified to the species level, which was in line with several previous studies evaluating the MicroScan panels [14,15,16]. The performance of MicroScan for Gram-negatives was similar or better than this of API^®^ (95.2%), Phoenix™ system from Becton–Dickinson (Franklin Lakes, NJ, USA) (92.5%), or Vitek^®^ 2 from bioMérieux (Marcy-l’Etoile, France) (88.6%) as reported by other studies [17,18].

The Gram-positive panel had a lower performance with 85.9% of tested isolates correctly identified up to species level. This was again in line with other published studies for *Staphylococcus* and *Enterococcus* species. There were no previous publications that reported the accuracy of MicroScan panels for *Streptococcus* species. Performance of MicroScan for Gram-positive isolates was lower than the reported performance of API^®^ (92.6%), Phoenix™ (93.6% accuracy), or Vitek^®^ 2 (90.2%) [17,19]. All *Staphylococcus aureus* isolates were correctly identified in our study, but *Streptococcus* species were identified less accurately, generating clinically relevant misidentifications for *Streptococcus pneumoniae* and, to a lesser extent, *Streptococcus anginosus*. Overall, 7.4% of the isolates tested would need additional testing; this was needed more often for Gram-positive than Gram-negative isolates.

Compared to conventional phenotypic testing as it is still conducted in most LRS, MicroScan performed considerably better—a study in Nigeria on 145 Enterobacterales isolates, comparing MALDI-TOF to conventional testing, found only 57.2% accuracy on genus level and 33.1% on species level [20].

### 4.2. Testing Isolates Not Represented in the Database, Robustness Testing

When inoculated on the MicroScan panels, isolates not represented in the database were misidentified with high probability scores in half of the cases, and only in a minority of them (6.1%), the user was alerted about the impossibility of identification. Likewise, testing on inappropriate panels (Gram-positive isolates on the Gram-negative panel and vice versa) or using an incorrect algorithm regularly led to incorrect identification with high probabilities instead of the expected “no identification possible” result. By contrast, errors in interpretation of beta-hemolysis did not impact the identification of hemolytic *Streptococcus* species.

The experiences of misidentifications at robustness testing highlight the importance of accurate performance and interpretation of orienting tests such as Gram stain or “spot tests” such as catalase and oxidase [21,22]. This poses challenges in LRS, as laboratory staff is frequently not familiar with clinical bacteriology [5] and the Gram stain is error-prone in inexperienced hands [23,24,25,26,27]. Apart from training, supervision, and guidance by the laboratory information support system, careful selection and quality control of orienting tests is needed, as was shown by the present experience with oxidase brands. Based on the present study (Table 5), identification results such as *Rhodococcus equi* and *Providencia rustigiannii* (both very rare in clinical bacteriology) should raise alerts to incorrect identifications.

### 4.3. Comparison between Automated and Visual Reading, Repeatability and Inter-Observer Agreement

Visual reading was slightly more accurate than reading by the autoSCAN-4 reader, except for *Streptococcus* species. The species agreement of 87.5%, was, however, lower than the 95% reported for the previous version of the reader (autoSCAN-3) [28]. No studies reporting agreement between visual reading and autoSCAN-4 reader have been published, although some studies showed excellent accuracy of autoSCAN-4 for Gram-negative bacteria [29,30]. Agreement between repetitions was higher with automated reading, which was not unexpected given the subjectivity in the reading of certain wells. Repeatability was poor for *Streptococcus* species.

### 4.4. Adaptation to LRS: Stability, Ease-of-Use

The temperature stability of the MicroScan panels, currently assured up to 25 °C, does not fulfill the requirements for tropical settings because cool storage (<30 °C) is not always feasible [31]. The shelf life of the reconstituted VP2 is too short, particularly for laboratories processing small amounts of samples. In addition, the 95% ethanol needed for reconstitution is often not easy to find in LRS.

As to ease of use, strong points are the MicroScan inoculation system. On the downside, visual reading of panels was considered complicated, poorly amenable to a swift work-flow, and error-prone. In the absence of the automated reader, the dedicated prototype microplate viewer (allowing quick change from white to the black background) considerably facilitated the reading process. The customized panels provided all tests on one panel; however, Gram stain results are still needed for reading and interpretation. Added value was therefore considered limited.

The Flesch–Kincaid Grade Level scores of the IFU indicated that a high level of schooling is required to understand the IFU. For patient leaflets in LRS, Flesch–Kincaid levels below 6 are desirable [13,32]. Although the language used in professional documents may be more complex, it should be reminded that the IFU will often not be available in the user’s native language, therefore low complexity is still preferable.

To obtain identification results, the user must purchase the LabPro software or use the (freely available) online Biotype Lookup Tool. For results with low probability scores, confirmatory tests are proposed by the software; however, many of these tests are unlikely to be available in LRS (Table 7).

### 4.5. Recommendations for Use and Further Development of the MicroScan System

Within the Mini-Lab project, the current limitations of the panels and IFU will be tackled by training, adapted laboratory procedures, bench aids, and the microplate viewer for visual reading. The Mini-lab project will further use the present results to create an expert decision support system that will anticipate potential errors such as those related to Gram stain interpretation. These expert rules will be made accessible outside MSF. In addition, we encourage the manufacturer to mitigate to the best possible extent the risks described above. To build in an extra fail-safe for Gram stain errors, the development of control wells for Gram-positive, Gram-negative, and yeast growth (consisting of vancomycin, colistin, and amphotericin B, respectively) has been proposed to the manufacturer. We further suggest simplifying the IFU and harmonize it with packaging. Bench aids and improved worksheets could be included with the product. Accuracy can further be improved over time by adding more LRS pathogens and contaminants to the database. Lastly, extended shelf-life testing and stability testing in tropical environments are necessary to assure product quality in LRS.

### 4.6. Strengths and Limitations of the Study

To our knowledge, this is the first study evaluating the MicroScan system on clinical isolates from LRS and the largest evaluating both Gram-positive and Gram-negative organisms, among which typical LRS pathogens [33,34]. Furthermore, we assessed robustness and ease of use. Robustness testing of in vitro diagnostics is encouraged to anticipate end users’ challenges, particularly in LRS [35,36]. To our knowledge, other commercial phenotypic identification systems have not yet been tested for robustness.

Because we did not dispose of Biosafety Level-3 (BSL-3) facilities, we could not test pathogens such as *B. pseudomallei* and *Brucella* species. Laboratory technicians were not blinded to the isolate’s identification when interpreting results; however, we do not believe this led to bias in the interpretation of test wells because they were unaware of which tests were supposed to be positive for which species. Lastly, this evaluation was performed in a reference laboratory in optimal conditions, by experienced laboratory technicians, and results can therefore not be extrapolated to a field setting. Performance and usability should be evaluated prospectively in LRS settings by the intended end user.

## 5. Conclusions

Challenged with clinical isolates from LRS, MicroScan Dried Overnight Identification panels had excellent performance for Gram-negative organisms. For important Gram-positive pathogens, accuracy ranged from excellent (*S. aureus*) to fair (*S. pneumoniae* and *S. anginosus*). The study further identified potential improvements in stability, robustness, and ease of use to assure adaptation of the MicroScan system to the constraints of LRS, particularly outside the MSF Mini-lab setting.

## Figures and Tables

**Table 1:** Test wells on the MicroScan Gram-positive and Gram-negative panels (Dried Overnight Positive ID Type 3 (PID3) and Dried Overnight Negative ID Type 2 (NID2), respectively) or embedded in the customized MSF panel (MSFNPID1) and reagents to be added. Underlined substrates are wells for which coverage with mineral oil before incubation is needed after inoculation. Substrates in bold are tests for which additional reagents need to be added after incubation and before reading.

Substrate.	Abbreviation	Panel	Substrate	Abbreviation	Panel
Glucose	GLU	Gram-negative	Lactose	LAC	Gram-positive
Sucrose	SUC	Gram-negative	Trehalose	TRE	Gram-positive
Inositol	INO	Gram-negative	Mannose	MNS	Gram-positive
Adonitol	ADO	Gram-negative	Ribose	RBS	Gram-positive
Rhamnose	RHA	Gram-negative	Inulin	INU	Gram-positive
Melibiose	MEL	Gram-negative	Mannitol	MAN	Gram-positive
Penicillin G 4 µg/mL	P4	Gram-negative	PNP-β-d-Glucuronide	PGR	Gram-positive
Kanamycin 4 µg/mL	K4	Gram-negative	PNP-β-d-Galactopyranoside	PGT	Gram-positive
Colistin 4 µg/mL	Cl4	Gram-negative	Indoxyl Phosphatase	IDX	Gram-positive
Cephalothin 8 µg/mL	Cf8	Gram-negative	Phosphatase	PHO	Gram-positive
Nitrofurantoin 64 g/mL	Fd64	Gram-negative	**Pyrrolidonyl-B-naphtylamide**	**PYR**	Gram-positive
Tobramycin 4 g/mL	To4	Gram-negative	Bile-Esculin	BE	Gram-positive
Cetrimide	CET	Gram-negative	Pyruvate	PRV	Gram-positive
Lysine	LYS	Gram-negative	Bacitracin	BAC	Gram-positive
Ornithine	ORN	Gram-negative	Crystal Violet	CV	Gram-positive
**Tryptophan Deaminase**	**TDA**	Gram-negative	Bacitracin 0.05 g/mL	MS	Gram-positive
Esculin	ESC	Gram-negative	Novobiocin 1.6 g/mL	NOV	Gram-positive
o-Nitrophenyl-d-Galactopyranoside	ONPG	Gram-negative	Optochin	OPT	Gram-positive
Citrate	CIT	Gram-negative	6.5% NaCl	NACL	Gram-positive
Malonate	MAL	Gram-negative	Sorbitol	SOR	Both panels
Acetamide	ACE	Gram-negative	Raffinose	RAF	Both panels
Tartrate	TAR	Gram-negative	Arabinose	ARA	Both panels
Oxidation Base Control	OF/B	Gram-negative	Arginine	ARG	Both panels
Oxidation of glucose	OF/G	Gram-negative	**Nitrate**	**NIT**	Both panels
Hydrogen Sulfide	H_2_S	Gram-negative	**Voges-Proskauer**	**VP**	Both panels
**Indole**	**IND**	Gram-negative	Urea	URE	Both panels
Decarboxylase Base Control	DCB	Gram-negative			

**Table 2:** Overall performance of the MicroScan Gram-negative, Gram-positive, and MSF ID panels for species represented in the MicroScan database. N = total number of isolates tested. Details of incorrect identifications with their probability scores are listed in Table 3. * Formerly part of *Streptococcus bovis* group.

Species	N	Correct Identification		Incorrect Identification		Identification Not Possible
		High Probability Score	Low Probability Score	High Probability Score	Low Probability Score	
**Enterobacterales**						
*Escherichia coli*	28	24	3	-	1	-
*Klebsiella pneumoniae*	13	13	-	-	-	-
*Klebsiella oxytoca*	3	3	-	-	-	-
*Enterobacter cloacae* complex	15	14	-	-	1	-
*Citrobacter freundii* complex	9	9	-	-	-	-
*Kluyvera ascorbata*	1	1	-	-	-	-
*Salmonella* Typhi	10	10	-	-	-	-
*Salmonella* Paratyphi A	10	9	-	1	-	-
*Salmonella* Typhimurium	10	10	-	-	-	-
*Salmonella* Choleraesuis	9	8	-	-	1	-
*Shigella* species	10	9	-	-	1	-
*Morganella morganii*	4	4	-	-	-	-
*Proteus mirabilis*	7	7	-	-	-	-
*Providencia rettgerii*	1	1	-	-	-	-
**Enterobacterales**	130	122 (93.8%)	3 (2.3%)	1 (0.8%)	4 (3.1%)	0 (0%)
**Non-fermenting Gram-negative organisms and *Aeromonas/Vibrio* species**						
*Pseudomonas aeruginosa*	11	11	-	-	-	-
*Acinetobacter baumannii*	9	8	-	1	-	-
*Achromobacter xylosoxidans*	10	8	-	1	1	-
*Burkholderia cepacia*	15	13	1	-	1	-
*Stenotrophomonas maltophilia*	15	14	1	-	-	-
*Aeromonas species*	3	2	-	-	1	-
*Vibrio alginolyticus*	2	2	-	-	-	-
**Non-fermenters**	65	58 (89.2%)	2 (3.1%)	2 (3.1%)	3 (4.6%)	0 (0%)
**Total Gram-negative isolates**						
**Total Gram-negative**	195	180 (92.3%)	5 (2.6%)	3 (1.5%)	7 (3.6%)	-
**Species**	**N**	**Correct Identification**		**Incorrect Identification**		**Identification Not Possible**
		**High Probability Score**	**Low Probability Score**	**High Probability Score**	**Low Probability Score**	
**Micrococcaceae**						
*Staphylococcus aureus*	14	14	-	-	-	-
*Staphylococcus hominis*	3	2	1	-	-	-
*Staphylococcus epidermidis*	11	10	-	1	-	-
*Staphylococcus lugdunensis*	3	3	-	-	-	-
*Staphylococcus lentus*	1	-	-	1	-	-
*Staphylococcus haemolyticus*	6	3	-	2	1	-
*Listeria monocytogenes*	2	2	-	-	-	-
*Micrococcus* species	4	4	-	-	-	-
**Total Staphylococcus/Micrococcus**	44	38 (86.4%)	1 (2.3%)	4 (9.1%)	1 (2.3%)	0 (0%)
**Streptococcaceae**						
*Enterococcus faecalis*	7	7	-	-	-	-
*Enterococcus faecium*	7	4	-	2	-	1
*Enterococcus casseliflavus*	1	-	-	1	-	-
*Streptococcus agalactiae*	13	11	1	-	1	-
*Streptococcus anginosus*	8	2	2	3	-	1
*Streptococcus pneumoniae*	12	9	-	2	1	-
*Streptococcus gallolyticus* *	10	9	1	-	-	-
*Streptococcus lutetiensis* *	1	-	-	-	1	-
*Streptococcus pyogenes*	13	13	-	-	-	-
*Streptococcus dysgalactiae* (group C & G)	6	6	-	-	-	-
*Streptococcus mitis*	5	2	3	-	-	-
*Streptococcus salivarius*	1	1	-	-	-	-
**Total *Streptococcus/Enterococcus* species**	84	64 (76.2%)	7 (8.3%)	8 (9.5%)	3 (3.6%)	2 (2.4%)
**Total Gram-positive isolates**						
**Total Gram-positive**	128	102 (79.7%)	8 (6.3%)	12 (9.4%)	4 (3.9%)	2 (1.6%)
**Overall total**	323	282 (87.3%)	13 (4.0%)	15 (4.6%)	11 (3.4%)	2 (0.6%)

**Table 3:** Overall performance of the MicroScan Gram-negative and Gram-positive panels for micro-organisms not represented in the MicroScan database. All identifications by the MicroScan system are by definition incorrect because the species are not represented in the database. The expected result for these species is “identification not possible”. N = total number of isolates tested. Overall, * 44 different isolates were tested, five of which on both the Gram-positive as the Gram-negative panel.

Species	N	Identification Not Possible	Low Probability Identification	High Probability Identification
Gram-positive bacilli (*Bacillus* & *Corynebacterium* species)	16	1	8	7
*Streptococcus suis*	11	-	5	6
*Burkholderia* species (non-cepacia, non-pseudomallei)	8	2	2	4
Yeast species (on Gram-positive panel)	9	-	-	9
Yeast species (on Gram-negative panel)	5	-	5	-
Total (species not represented in MicroScan database)	49 *	3 (6.1%)	20 (40.8%)	26 (53.1%)

**Table 4:** Details of incorrectly identified isolates. Potential clinical relevance is also indicated; limited clinical relevance means that the two pathogens are similar in clinical, epidemiological, and infection control aspects. Probability scores are reported by the MicroScan LabPro software with two decimals. A probability score of ≥85.00% is considered as “high probability”. N = total number of isolates tested. * As identified by matrix-assisted laser desorption/ionization time-of-flight mass spectrometry (MALDI-TOF) (and serotyping when necessary).

Species/Serotype *	Nr. of Isolates with Incorrect Identification/N	Incorrect Result by MicroScan	Probability Score	Clinical Relevance of Error
**Gram-negative species**				
*Escherichia coli*	1/28	*Escherichia fergusonii*	67.88%	Limited
*Enterobacter cloacae*	1/15	*Enterobacter amnigenus*	58.26%	Limited
*Salmonella* Paratyphi A	1/10	*Salmonella* Typhi	98.87%	Moderate: pathogens are clinically similar, but distinction has epidemiological relevance
*Salmonella* Choleraesuis	1/9	*Salmonella* Typhi	57.06%	High: pathogens are both clinically and epidemiologically distinct
*Shigella* species	1/10	*Klebsiella ozaenae*	72.69%	High: pathogens have different clinical presentations; *Shigella* infection is of high epidemiological importance (outbreak potential)
*Acinetobacter baumannii*	1/9	*Acinetobacter lwoffii*	92.88%	Moderate: pathogens may have different clinical presentation
*Achromobacter xylosoxidans*	2/10	*Rhizobactrum radiobacter* *Burkholderia cepacia*	49.88% 94.19%	Limited
*Aeromonas caviae*	1/3	*Aeromonas hydrophila*	61.71%	Limited
**Gram-positive species**				
*Staphylococcus epidermidis*	1/11	*Staphylococcus hyicus*	91.14%	Limited
*Staphylococcus lentus*	1/1	*Staphylococcus sciuri*	96.73%	Limited
*Staphylococcus haemolyticus*	3/6	*Staphylococcus lugdunensis* *Staphylococcus lugdunensis*	53.67% 93.66%	Moderate: pathogens may have different clinical presentation
*Enterococcus faecium*	2/7	*Enterococcus raffinosus* *Enterococcus durans*	95.39% 99.68%	Moderate: pathogens have different clinical significance
*Enterococcus casseliflavus*	1/1	*Enterococcus gallinarum*	59.68%	Limited: both are associated with increased vancomycin resistance
*Streptococcus anginosus*	3/8	*Streptococcus mitis/oralis* *Streptococcus salivarius* *Streptococcus parasanguinis*	86.99% 88.56% 99.99%	High: *Streptococcus anginosus* is associated with purulent infections; other viridans streptococci are more likely contaminants or implicated in infectious endocarditis
*Streptococcus pneumoniae*	3/12	*Rhodococcus equi* *Gemella* species *Gemella* species	99.99% 51.48% 95.79%	High: *Streptococcus pneumoniae* is a more common pathogen than *Rhodococcus equi* or *Gemella* and clinical presentation is very different
*Streptococcus agalactiae*	1/13	*Streptococcus dysgalactiae*	99.99%	Limited; except for screening in pregnancy

**Table 5:** Most frequent incorrect identifications with high probability score for species not represented in the database or “incorrect” use of the panels (Gram-positive isolates inoculated on Gram-negative panels and vice versa, Streptococcus using Micrococcus/Staphylococcus algorithm and vice versa). N = total number of isolates tested.

Species.	Algorithm Used (for Gram-Positive Panels)	N	Number of Isolates with this Result	Result
**Species not represented in the database**				
Yeast species	Micrococcus	9	9	*Staphylococcus cohnii*
	Streptococcus		9	*Rhodococcus equi*
*Corynebacterium* species	Micrococcus	6	1	*Micrococcus species*
	Streptococcus	6	1	*Rhodococcus equi*
*Bacillus* species	Micrococcus	10	4	*Staphylococcus auricularis*
**Gram-positive species on Gram-negative panel**				
*Streptococcus dysgalactiae*		2	2	*Providencia rustigiannii*
*Streptococcus suis*		10	9	*Providencia rustigiannii*
*Staphylococcus lentus*		1	1	*Providencia rustigiannii*
*Streptococcus agalactiae*		5	1	*Providencia rustigiannii*
**Gram-negative species on Gram-positive panel**				
*Escherichia coli/paracoli*	Streptococcus	10	3	*Rhodococcus equi*
*Acinetobacter baumannii*	Micrococcus	4	3	*Staphylococcus cohnii*
	Micrococcus		1	*Staphylococcus auricularis*
	Streptococcus		1	*Rhodococcus equi*
*Stenotrophomonas maltophilia*	Streptococcus	4	2	*Rhodococcus equi*
*Burkholderia cepacia*	Streptococcus	4	3	*Rhodococcus equi*
*Burkholderia thailandensis*	Streptococcus	4	1	*Rhodococcus equi*
*Burkholderia ubonensis*	Streptococcus	1	1	*Rhodococcus equi*
**Gram-positive species using incorrect algorithm on Gram-positive panel**				
*Staphylococcus aureus*	Streptococcus	14	3	*Rhodococcus equi*
*Micrococcus* species	Streptococcus	4	2	*Rhodococcus equi*
*Staphylococcus lentus*	Streptococcus	1	1	*Rhodococcus equi*
*Streptococcus gallolyticus*	Micrococcus	10	9	*Listeria monocytogenes*
*Streptococcus pneumoniae*	Micrococcus	12	9	*Micrococcus species*
			2	*Staphylococcus auricularis*
*Streptococcus pyogenes*	Micrococcus	13	2	*Staphylococcus auricularis*
*Streptococcus agalactiae*	Micrococcus	13	1	*Staphylococcus auricularis*
*Streptococcus anginosus*	Micrococcus	8	1	*Staphylococcus auricularis*
*Streptococcus mitis/oralis*	Micrococcus	5	1	*Staphylococcus auricularis*

**Table 6:** Results when isolates were tested on an incorrect panel or using an incorrect algorithm, i.e., inoculation of Gram-negative species on Gram-positive panels or vice versa, or of Gram-positive species incorrectly assigned as “Streptococcus” or “Micrococcus” based on faulty catalase or microscopy results. The expected result for this type of incorrect panel use is “identification not possible”. For Gram-negative species inoculated on Gram-positive panel, we assumed Micrococcus interpretation for the results in this table, as most of them would be catalase positive. Note that all results other than “identification not possible” are wrong; detailed results for each species can be found in Supplementary Table S3. Incorrect identifications with high probability are listed in Table 5. N = total number of isolates tested.

Species.	N	Panel/Algorithm Used	Number “Identification not Possible” (%)	Number Low Probability Identification (%)	Number High Probability Identification (%)
**Gram-negative species tested on Gram-positive panels and vice versa**					
Enterobacterales	41	Gram-positive	27	9	5
Non-fermenters	23		10	6	7
**Total Gram-negatives on Gram-positive panel**	64		37 (57.8%)	15 (23.4%)	12 (18.8%)
*Staphylococcus* species	16	Gram-negative	1	11	4
*Streptococcus* species	53		15	12	26
Gram-positive bacilli (*Bacillus* & *Corynebacterium* species)	11		2	4	5
**Total Gram-positive on Gram-negative panel**	80		18 (22.5%)	27 (33.8%)	35 (43.8%)
***Staphylococcus*/*Micrococcus* species using *Streptococcus* species algorithm and vice versa**					
*Staphylococcus/Micrococcus* species and Gram-positive rods	60	*Streptococcus* algorithm	35	11	14
*Streptococcus/Enterococcus* species	95	*Micrococcus* algorithm	27	24	44
**Total Gram-positive with incorrect algorithm**	155		62 (40.0%)	35 (22.6%)	58 (37.4%)
**Total tested on incorrect panel/incorrect algorithm**	299		117 (39.1%)	77 (25.8%)	105 (35.1%)

**Table 7:** Ease-of-use assessment with a focus on use in low resource settings of different aspects of the MicroScan identification panels. Abbreviations: IFU = instructions for use; PEP = peptidase; PYR = pyrrolidonyl-b-naphtylamide; VP = Voges–Proskauer test.

Favorable Observations	Unfavorable Observations
**Storage of panels and reagents**	
Long shelf life of the panels and of most reagents	VP2 reagent must be reconstituted with 95% ethanol (not provided); after reconstitution, vial is stable for only 2 weeks
Panels and some reagents can be stored at room temperature	Many reagents (nitrate, VP, ferric chloride, Kovac’s) require storage at 2–8 °C
**Instructions for use, labeling, and packaging**	
Panels are packaged separately with desiccant, (StripPax^®^, Multisorb Technologies, New York, NY, USA)	Reconstitution of VP2 reagent not explained in IFU, only on box of reagent
Sturdy outer packaging	
IFU were delivered at request by the distributor	IFU are not delivered in the box with the products; they are available online but not easy to find
	Flesch Kincaid Grade level of IFU was 9.14 for the Gram-negative IFU, 11.06 for the Gram-positive IFU and 10.30 for the MSF panel IFU; these levels point to a high level of education needed to comprehend the text
	Instructions in IFU for interpretation of negative/positive wells are often ambiguous.e.g., yellow to orange color for carbohydrate tests must be considered positive, whereas orange to red must be considered negative
Reagent bottles are labeled with the test name: easy to add right reagent to right test well	Use of reagent names is inconsistent between IFU and reagent bottles (e.g., 5% alpha naphtol in IFU, VP2 on bottle)
	The PYR test reagent has “PEP” on the bottle and not “PYR”
	Limited space for writing patient identification and sample number on the panel
**Inoculation and reading**	
Most panels could be inoculated using the Prompt™ system	Prompt™ is not advised for use in slow-growing *Streptococcus* species; we repeated testing of 14 *Streptococcus* isolates with turbidity standard method due to incorrect results with Prompt™; 50% of these had a correct result with turbidity standard method
Prompt ™ was easy to use and time-efficient	
RENOK system was easy to use and time-efficient	Large amounts of plastic waste associated with the RENOK system
	Difficulties in disposing of waste without spillage
	Difficulties in incubation of panels without spillage
Favorable observations	**Unfavorable observations**
IFU recognizes that interpretation of test results requires trained clinical staff	Visual reading of panels was considered difficult as positivity of test wells was often doubtful
Automated reading was more user-friendly as it removed subjectivity and eliminated clerical errors	autoSCAN-4 does not indicate automatically that panels must be re-incubated
	autoSCAN-4 reader dimensions are very large: 48.5 cm × 24 cm × 58 cm
Prototype microplate viewer (JP Selecta) with magnifier and interchangeable white/black background considerably facilitated the reading	Both white and black background needed for reading of wells
	Reagents can be added only after visual assessment of certain wells indicating the need for re-incubation; panels must be re-incubated if specific combinations of wells are negative—there are 6 of these combinations that consist each of 1 to 3 wells unevenly distributed on the panels
	Different reading times for different reagents
	Re-incubation was required for 15.3% of panels in our study; 13.3% (27/203) of Gram-negative isolates (mostly non-fermenters) and 17.7% (29/164) of Gram-positive isolates (mainly *Streptococcus* isolates).
**Software and Biotype Lookup Tool**	
LabPro software and reader easy to use	Beckman Coulter worksheets not delivered with the panels; available online but hard to find
Calibration and QC of reader are alerted by the reader automatically and do not require user input	Test position on worksheet different from test position on the panel (for Gram-negative panel) (Figure 5).
Biotype Lookup Tools freely available online	
Both the online Biotype Lookup Tool and LabPro software propose additional tests to confirm low-probability identification	Many of the confirmatory tests proposed by the software are unlikely to be easily available in LRS Examples of recommended confirmatory tests: *E. coli*: cellobiose fermentation, growth in potassium cyanide and Christensen urea agar *Salmonella* Typhi: trehalose fermentation, serotyping *S. pneumoniae*: leucine aminopeptidase and bile solubility *S. anginosus*: beta-d-acetylgalactosaminidase, alpha-glucosidase, beta-glucosidase, hyaluronidase, neuraminidase

## Data Availability

The database for this manuscript will be made open access. Access requests for ITM research data can be made to ITM’s central point for research data access by means of submitting the completed Data Access Request Form. These requests will be reviewed for approval by ITMs Data Access Committee (https://www.itg.be/E/data-sharing-open-access).

## Supplementary Materials

The following are available online at https://www.mdpi.com/2075-4418/11/2/349/s1, Table S1: Lot numbers of tested panels, Table S2: Geographical origin of the isolates, Table S3: Detailed results for incorrect use of the panels, Table S4: Agreement between visual and automated (autoSCAN-4) reading, Table S5: Results for inter-observer agreement between two independent readers who read the panels visually.

## References

[1] Ombelet S., Ronat J.B., Walsh T., Yansouni C.P., Cox J., Vlieghe E., Martiny D., Semret M., Vandenberg O., Jacobs J. (2018). Clinical bacteriology in low-resource settings: Today’s solutions. Lancet Infect. Dis..

[2] Petti C.A., Polage C.R., Quinn T.C., Ronald A.R., Sande M.A. (2006). Laboratory Medicine in Africa: A Barrier to Effective Health Care. Clin. Infect. Dis..

[3] The European Committee on Antimicrobial Susceptibility Testing (2020). Breakpoint Tables for Interpretation of MICs and Zone Diameters. Version 10.0. http://www.eucast.org.

[4] CLSI (2020). M100: Performance Standards for Antimicrobial Susceptibility Testing.

[5] Jacobs J., Hardy L., Semret M., Lunguya O., Phe T., Affolabi D., Yansouni C., Vandenberg O. (2019). Diagnostic Bacteriology in District Hospitals in Sub-Saharan Africa: At the Forefront of the Containment of Antimicrobial Resistance. Front. Med..

[6] Franco-Duarte R., Černáková L., Kadam S., Kaushik K.S., Salehi B., Bevilacqua A., Corbo M.R., Antolak H., Dybka-Stępień K., Leszczewicz M. (2019). Advances in chemical and biological methods to identify microorganisms—From past to present. Microorganisms.

[7] Leber A.L. (2016). Clinical Microbiology Procedures Handbook.

[8] Jorgensen J.H., Jorgensen J.H., Carroll K.C., Funke G., Pfaller M.A. (2015). Manual of Clinical Microbiology.

[9] Sandle T. (2016). Microbial identification. Pharmaceutical Microbiology.

[10] Natale A., Ronat J.-B., Mazoyer A., Rochard A., Boillot B., Hubert J., Baillet B., Ducasse M., Mantelet F., Oueslati S. (2020). The Mini-Lab: Accessible clinical bacteriology for low-resource settings. Lancet Microbe.

[11] Russell F.M., Biribo S.S.N., Selvaraj G., Oppedisano F., Warren S., Seduadua A., Mulholland E.K., Carapetis J.R. (2006). As a bacterial culture medium, citrated sheep blood agar is a practical alternative to citrated human blood agar in laboratories of developing countries. J. Clin. Microbiol..

[12] Zomorodian K., Javad M., Safaei A., Bazargani A. (2011). Analysis of beta-hemolysis in human blood agars by Streptococcus pyogenes Analysis of beta-hemolysis in human blood agars by Streptococcus pyogenes. J. Microbiol. Methods.

[13] WHO (2017). Technical Guidance Series (TGS) for WHO Prequalification—Diagnostic Assessment Guidance on Test Guidance on Test Method Validation for In Vitro Diagnostic Medical Devices TGS–4.

[14] Jin W.Y., Jang S.J., Lee M.J., Park G., Kim M.J., Kook J.K., Kim D.M., Moon D.S., Park Y.J. (2011). Evaluation of VITEK 2, MicroScan, and Phoenix for identification of clinical isolates and reference strains. Diagn. Microbiol. Infect. Dis..

[15] Rhoads S., Marinelli L., Imperatrice C.A., Nachamkin I. (1995). Comparison of MicroScan WalkAway system and Vitek system for identification of gram-negative bacteria. J. Clin. Microbiol..

[16] Snyder J.W., Munier G.K., Johnson C.L. (2008). Direct comparison of the BD phoenix system with the MicroScan WalkAway system for identification and antimicrobial susceptibility testing of Enterobacteriaceae and nonfermentative gram-negative organisms. J. Clin. Microbiol..

[17] Chatzigeorgiou K.S., Sergentanis T.N., Tsiodras S., Hamodrakas S.J., Bagos P.G. (2011). Phoenix 100 versus Vitek 2 in the identification of gram-positive and gram-negative bacteria: A comprehensive meta-analysis. J. Clin. Microbiol..

[18] O’Hara C.M. (2005). Manual and Automated Instrumentation for Identication of Enterobacteriaceae and Other Aerobic Gram-Negative Bacilli. Clin Microbiol Rev.

[19] Von Baum H., Klemme F.R., Geiss H.K., Sonntag H.G. (1998). Comparative evaluation of a commercial system for identification of gram-positive cocci. Eur. J. Clin. Microbiol. Infect. Dis..

[20] Jesumirhewe C., Ogunlowo P.O., Olley M., Springer B., Allerberger F., Ruppitsch W. (2016). Accuracy of conventional identification methods used for Enterobacteriaceae isolates in three Nigerian hospitals. PeerJ.

[21] Baron E.J. (2001). Rapid Identification of Bacteria and Yeast: Summary of a National Committee for Clinical Laboratory Standards Proposed Guideline. Clin. Infect. Dis..

[22] Baron E.J., York M.K., Ferraro M.J., Rex J.H., Body B.A., Forbes B.A., Poole F.M., Sahm D.F., Tenover F.C., Turnidge J.D. (2008). M35-A2 Abbreviated Identification of Bacteria and.

[23] Rand K.H., Tillan M. (2006). Errors in interpretation of gram stains from positive blood cultures. Am. J. Clin. Pathol..

[24] Samuel L.P., Balada-Llasat J.M., Harrington A., Cavagnolo R. (2016). Multicenter assessment of gram stain error rates. J. Clin. Microbiol..

[25] Munson E., Block T., Basile J., Hryciuk J.E., Schell R.F. (2007). Mechanisms to assess Gram stain interpretation proficiency of technologists at satellite laboratories. J. Clin. Microbiol..

[26] (2013). Chandler, L. Challenges in Clinical Microbiology Testing.

[27] Yuan S., Astion M.L., Schapiro J., Limaye A.P. (2005). Clinical impact associated with corrected results in clinical microbiology testing. J. Clin. Microbiol..

[28] Ellner P.D., Myers D.A. (1981). Preliminary evaluation of the autoSCAN-3, an instrument for automated reading and interpretation of microdilution trays: Identification of aerobic gram-negative bacilli. J. Clin. Microbiol..

[29] Rhoden D.L., Smith P.B., Baker C.N., Schable B. (1985). autoSCAN-4 system for identification of gram-negative bacilli. J. Clin. Microbiol..

[30] Gavini F., Husson M.O., Izard D., Bernigaud A., Quiviger B. (1988). Evaluation of Autoscan-4 for identification of members of the family Enterobacteriaceae. J. Clin. Microbiol..

[31] World Health Organization—R&D Blueprint (2020). COVID-19 Target Product Profiles for Priority Diagnostics to Support Response to the COVID-19 Pandemic v.0.1.

[32] Gillet P., Maltha J., Hermans V., Ravinetto R., Bruggeman C., Jacobs J. (2011). Malaria rapid diagnostic kits: Quality of packaging, design and labelling of boxes and components and readability and accuracy of information inserts. Malar. J..

[33] Reddy E.A., Shaw A.V., Crump J.A. (2010). Community-acquired bloodstream infections in Africa: A systematic review and meta-analysis. Lancet Infect. Dis..

[34] Deen J., von Seidlein L., Andersen F., Elle N., White N.J., Lubell Y. (2012). Community-acquired bacterial bloodstream infections in developing countries in south and southeast Asia: A systematic review. Lancet Infect. Dis..

[35] WHO (2020). Technical Specification Series. https://www.who.int/diagnostics_laboratory/guidance/technical-specifications-series/en/.

[36] Gerth-Guyette E., Malacad C.C., Demonteverde M.P., Faulx D., Lochhead M.J., Lupisan S.P., Leader B.T., Tallo V.L. (2018). Understanding user requirements to improve adoption of influenza diagnostics in clinical care within Metro Manila. Health Sci. Rep..

