# Supplementary material for: Evaluation of MicroScan Bacterial Identification Panels for Low-Resource Settings
Source: Diagnostics (Basel). 2021 Feb 19;11(2):349. doi: 10.3390/diagnostics11020349 (PMC7922174; doi:10.3390/diagnostics11020349)
Supplement: Supplementary file 1 [file diagnostics-11-00349-s001.pdf]

Table S1. Lot numbers of tested panels.

	Lot number	Expiry date	Period used
**Gram-positive panels**	2019-04-24<br>2019-12-27<br><br>2020-07-02	24/4/2019<br>27/12/2019<br><br>2/7/2020	15/04/2019 – 19/04/2019<br>22/04/2019 – 03/05/2019<br>16/10/2019 – 6/12/2019<br>27/01/2020 – 31/01/2020
**Gram-negative panels**	2019-04-26<br>2019-11-30<br>2019-12-17<br>2020-03-01	26/4/2019<br>30/11/2019<br>17/12/2019<br>01/03/2020	11/03/2019 – 19/04/2019<br>22/04/2019 – 03/05/2019<br>16/10/2019 – 29/11/2019<br>2/12/2019 – 6/12/2019<br>06/01/2020 – 31/01/2020
**MSF ID panels**	2019-04-16	16/4/2019	18/03/2019 – 12/04/2019

Table S2. Geographical origin of the isolates. These isolates were obtained from ITM travel clinic and surveillance studies.

**Species**	**Total number of isolates tested**	**Africa**	**Asia**	**South America**	**Europe**	
					Clinical isolates	Reference strains
**Gram-negative species**						
Escherichia coli	28	24	4	-	-	-
Klebsiella pneumoniae	13	10	2	1	-	-
Klebsiella oxytoca	3	2	-	1	-	-
Enterobacter cloacae complex	15	12	-	3	-	-
Citrobacter freundii complex	9	8	1	-	-	-
Kluyvera ascorbata	1	1	-	-	-	-
Salmonella Typhi	10	6	3	1	-	-
Salmonella Paratyphi A	10	4	6	-	-	-
Salmonella Typhimurium	10	8	2	-	-	-
Salmonella Choleraesuis	9	-	8	1	-	-
Shigella species	10	4	-	-	-	6
Morganella morganii	4	4	-	-	-	-
Proteus mirabilis	7	4	3	-	-	-
Providencia rettgerii	1	1	-	-	-	-
Pseudomonas aeruginosa	11	6	3	2	-	
Acinetobacter baumannii	9	6	-	3	-	-
Achromobacter xylosoxidans	10	9	1	-	-	-
Burkholderia cepacia	15	14	-	1	-	-
Burkholderia thailandensis	6	-	6	-	-	-
Burkholderia ubonensis	1	-	1	-	-	-
Burkholderia oklahomensis	1	-	1	-	-	-
Stenotrophomonas maltophilia	15	14	1	-	-	-
Aeromonas species	3	-	3	-	-	-
Vibrio alginolyticus	2	-	-	-	-	2
**Gram-positive species**						
**Species**	**Total number of isolates tested**	**Africa**	**Asia**	**South America**	**Europe**	
					Clinical isolates	Reference strains
Staphylococcus aureus	14	10	4	-	-	-
Staphylococcus hominis	3	1	2	-	-	-
Staphylococcus epidermidis	11	9	-	-	-	2
Staphylococcus lugdunensis	3	1	-	-	-	2
Staphylococcus lentus	1	-	1	-	-	-

Staphylococcus haemolyticus	6	6	-	-	-	-
Listeria monocytogenes	2	-	-	-	-	2
Micrococcus species	4	3	1	-	-	-
Enterococcus faecalis	7	7	-	-	-	-
Enterococcus faecium	7	5	1	1	-	-
Enterococcus casseliflavus	1	-	1	-	-	-
Streptococcus agalactiae	13	1	4	-	2	6
Streptococcus anginosus	8	1	2	-	2	3
Streptococcus pneumoniae	12	10	-	-	2	
*Streptococcus gallolyticus**	10	1	6	-	-	3
*Streptococcus lutetiensis**	1	-	1	-	-	-
Streptococcus pyogenes	13	5	4	-	1	3
Streptococcus dysgalactiae (group C & G)	6	-	3	-	-	3
Streptococcus mitis/oralis	5	4	1	-	-	-
Streptococcus salivarius	1	-	1	-	-	-
Bacillus species	10	10	-	-	-	-
Corynebacterium species	6	2	1	-	-	3
Yeast species	9	9	-	-	-	-
Streptococcus suis	11	-	11	-	-	-
**Total**	**367**	**222 (60.5%)**	**89 (24.3%)**	**14 (3.8%)**	**7 (1.9%)**	**35 (9.5%)**

* Formerly part of *Streptococcus bovis* group

Table S3. Detailed results for incorrect use of panels. Species not listed in the database are marked with an asterisk (*).

Species	Total number of isolates tested	Identification not possible	Incorrect low probability identifications	Incorrect high probability identifications
**Gram-positive isolates on Gram-negative panel**				
S. aureus	5	-	3	2
S. hominis	3	1	2	-
S. epidermidis	2	-	2	-
S. lentus	1	-	-	1
S. haemolyticus	1	-	1	-
***Micrococcus* species**	4	-	3	1
***Enterococcus* species**	10	6	1	3
S. agalactiae	6	2	-	4
S. anginosus	1	-	1	-
S. pneumoniae	3	-	2	1
S. pyogenes	4	3	1	-
S. dysgalactiae	4	1	1	2
S. oralis/mitis	4	-	2	2
S. suis	10	1	-	9
***Bacillus* species ***	8	2	3	3
***Corynebacterium* species ***	3	-	1	2
**Yeast species ***	5	-	5	-
**Staphylococcus interpreted with Streptococcus algorithm**				
S. aureus	14	8	-	6
S. hominis	3	2	-	1
S. epidermidis	11	9	2	-
S. lugdunensis	3	3	-	-
S. lentus	1	-	-	1
S. haemolyticus	6	6	-	-
Listeria monocytogenes	2	2	-	-

Micrococcus species	4	-	2	2
**Streptococcus interpreted with Staphylococcus algorithm**				
Enterococcus species	15	9	1	5
**Species**	**Total number of isolates tested**	**Identification not possible**	**Incorrect low probability identifications**	**Incorrect high probability identifications**
S. agalactiae	13	3	6	4
S. anginosus	8	1	3	4
S. pneumoniae	12	-	1	11
S. gallolyticus	10	-	-	10
S. lutetiensis	1	-	1	-
S. pyogenes	13	9	-	4
S. dysgalactiae	6	3	-	3
S. oralis/mitis	5	1	2	2
S. salivarius	1	-	1	-

Gram-negative species on Gram-positive panel

		**Identification not possible**		**Incorrect low probability identifications**		**Incorrect high probability identifications**	
		***Micrococcus* interpretation**	***Streptococcus* interpretation**	***Micrococcus* interpretation**	***Streptococcus* interpretation**	***Micrococcus* interpretation**	***Streptococcus* interpretation**
Escherichia coli	10	4	7	2	-	4	3
***Enterobacter cloacae* complex**	5	5	5	-	-	-	-
Kluyvera ascorbata	1	-	1	-	-	1	-
***Citrobacter freundii* complex**	5	4	5	1	-	-	-
***Salmonella* Typhi**	2	2	2	-	-	-	-
***Salmonella* Paratyphi A**	3	2	3	1	-	-	-
***Salmonella* Typhimurium**	5	-	5	5	-	-	-
***Salmonella* Choleraesuis**	5	5	5	-	-	-	-
***Shigella* species**	5	5	5	-	-	-	-
Pseudomonas aeruginosa	3	3	3	-	-	-	-
Acinetobacter baumannii	4	-	-	-	3	4	1
Achromobacter xylosoxidans	1	1	1	-	-	-	-
Burkholderia cepacia	4	2	1	2	-	-	3
Stenotrophomonas maltophilia	4	1	-	3	1	-	3
***Burkholderia thailandensis* ***	5	2	4	1	-	2	1
***Burkholderia oklahomensis* ***	1	1	1	-	-	-	-
***Burkholderia ubonensis* ***	1	-	-	-	-	1	1

Table S4. Agreement between visual and automated (autoSCAN-4) reading: two readings had species agreement if both readings resulted in the same result up to species level. Total biotype agreement indicates the same result was read for each well. A result was considered correct if it was accurate up to species level, disregarding the probability score. For species not represented in the database, correct identification was not possible, so these results were not included.

Species	Number of isolates tested	% species agreement	% biotype agreement	% correct identification	
				Automated	Visual

Enterobacterales	55	98.2%	60%	96.4%	98.2%
Non-fermenters (including *Aeromonas/Vibrio* species)	41	87.8%	58.5%	85.4%	92.7%
**Total Gram-negative tested**	**96**	**93.8%**	**59.4%**	**91.7%**	**95.8%**
Staphylococcus/ Micrococcus / Listeria isolates	23	91.3%	60.9%	90.5%	91.3%
Streptococcus / Enterococcus	41	87.8%	53.7%	80.5%	78.0%
Gram-positive rods (not represented in database)	9	77.8%	44.4%	-	-
Streptococcus suis (not represented in database)	9	11.1%	0%	-	-
**Total Gram-positive; only species represented in database**	**64**	**89.1%**	**56.3%**	**82.8%**	**81.3%**
Total	178	87.1%	54.5%	-	-
**Total including only species represented in database**	**160**	**91.9%**	**58.1%**	**87.5%**	**90.6%**

Table S5. Results for inter-observer agreement between two independent readers who read the panels visually. Two readings had species agreement if both readings resulted in the same result up to species level. Total biotype agreement indicates the same result was read for each well. P-value for difference in accuracy between visual and automated reading was calculated using McNemar test for paired samples. The species tested for repeatability can be found in Supplementary Table 4. The abbreviations for the tests are explained in footnote.

	**Number of isolates tested for inter-observer agreement**	**Number of isolates with categorical agreement (% of total)**	**Number of isolates with total biotype agreement (% of total)**	**Total number of wells read**	**Number of wells differently read by the observers**	**Disagreement of which wells was seen? ***	**Which well differences led to categorical disagreement?**
**Enterobacterales**	15	14 (93.3%)	8 (53.3%)	480	13	ARG; K4; RAF; ARA; NIT ; CL4 ; CET; CIT ; **VP x 3**; IND ; FD64	CL4
**Non-fermenting Gram-negative organisms**	8	8 (100%)	7 (87.5%)	256	1	SOR	/
Aeromonas caviae	1	1 (100%)	0	32	1	IND	/
***Staphylococcus* species**	8	8 (100%)	4 (50.0%)	208	5	NOV x 2; **VP x 2;** ARG	/
***Streptococcus* species**	18	18 (100%)	14 (77.8%)	468	4	**VP**; PHO; PRV; PYR	/
**Total**	50	49 (98.0%)	33 (66.0%)	1444	24		

* Abbreviations of tests: ARA = L-arabinose fermentation; ARG = arginine dehydrolase; CET = cetrimide resistance; CIT = citrate; CL4 = colistin resistance; FD64 = nitrofurantoin resistance; IND = indole; K4 = kanamycin resistance; NIT = nitrate reduction; NOV = novobiocin resistance; PHO = phosphatase; PRV = pyruvate utilization ; PYR = Pyrrolidonyl-β-naphtylamide; RAF = raffinose fermentation ; SOR = sorbitol fermentation; VP = Voges Proskauer.
